# Dermatologic Adverse Effects of Protein Supplements, Anabolic Performance Enhancers, and Other Performance Enhancers

**DOI:** 10.1111/jocd.71247

**Published:** 2026-10-22

**Authors:** Firas F. Haddad, Lina Abdullah, Mazen Kurban, Pierre Abi Akl, Ossama Abbas

**Affiliations:** ^1^ Department of Dermatology American University of Beirut Medical Center Hamra Beirut Lebanon; ^2^ Department of Otorhinolaryngology American University of Beirut Medical Center Hamra Beirut Lebanon

**Keywords:** dermatology, nutrition, sports supplements

## Abstract

**Background:**

The prevalence of consumption of sports supplements and performance enhancers has increased among the general population. While these products are intended to improve muscle growth, endurance, and performance, several dermatologic effects have been reported.

**Objective:**

To summarize the current evidence on cutaneous manifestations associated with commonly used sports supplements and anabolic agents.

**Results:**

Whey protein supplementation has been associated with acneiform eruptions, likely mediated through hormonal and metabolic pathways involving insulin and insulin‐like growth factor‐1. Observational studies and case series support this association, although randomized evidence remains limited and conflicting. Branched‐chain amino acid supplementation may contribute to insulin resistance, potentially leading to dermatologic signs such as acne, acanthosis nigricans, and skin tags, though data are scarce. Anabolic androgenic steroids are strongly linked to multiple cutaneous adverse effects, including acne vulgaris, severe acne variants, seborrhea, androgenetic alopecia, and other more rarely reported dermatologic adverse events. Other supplements—including beta‐alanine, selenium, niacin, zinc, herbal products, and high‐dose vitamins—have also been associated with various dermatologic reactions.

**Conclusion:**

Sports supplements may contribute to a range of dermatologic manifestations. Further prospective studies are needed to clarify causal relationships and better define the dermatologic risks associated with these widely used products.

## 1 Introduction

The consumption of several forms of nutritional supplementation is on the rise. These patients may present to dermatologic clinics with dermatoses that may be related to the consumption of these supplements [1]. Massive marketing on social media, widespread availability and ease of access, and promotion by gym instructors are contributing to this exponential rise [2]. Knowledge of these agents as well as possible cutaneous adverse events is essential for proper management of patients presenting with various dermatoses possibly related to the consumption of these products.

Physical activity promotes numerous anti‐inflammatory effects that may benefit the skin by reducing chronic inflammation, modulation of cytokine signaling, and improvements in metabolic homeostasis. Exercise induces the release of cytokines such as interleukin‐10 (IL‐10) that exerts an anti‐inflammatory effect, and reducing pro‐inflammatory signals such as tumor necrosis factor‐α (TNF‐α) and IL‐6 derived from adipose tissue [3]. In addition, exercise enhances anti‐oxidant defenses, improves insulin sensitivity, and mitigates oxidative stress. All these mechanisms may reduce the inflammation implicated in dermatologic diseases such as psoriasis and acne [3]. Vitamin D, frequently supplemented by athletes, also possesses immunomodulatory properties through regulation of innate and adaptive immune responses and has been investigated as an adjunctive factor in inflammatory dermatoses [4]. Collectively, these observations suggest that while exercise itself may promote an anti‐inflammatory physiologic milieu, the use of certain sports supplements and anabolic agents may independently contribute to adverse cutaneous manifestations through hormonal, metabolic, or immune‐mediated mechanisms. Studies are needed to better delineate the interplay between exercise, supplementation, and skin disease.

The rise of social media, and the ease of sharing information on digital platforms has had a significant impact on public perception [5, 6, 7]. There has been an exponential rise in content creation related to diet and dietary supplementation over the past decade, and this has altered how people gain access to such information [8]. While social media has greatly improved access to dietary and nutritional information, it has also facilitated the widespread dissemination of misinformation [9]. Platforms such as TikTok enable rapid distribution of nutrition‐related content, much of which may be misleading. In a recent analysis, only approximately one‐third of nutrition‐related TikTok posts were deemed accurate [10].

The prevalence of consumption of these products varies between studies and ranges between 35.6% and 76.6% [11, 12]. As such, it is important for healthcare professionals as a whole, and dermatologists in specific to be more aware of these adverse events, as knowledge of them is key to earlier diagnosis and proper management of these adverse events. The purpose of this review is to summarize and review the current published literature on the possible cutaneous adverse events of the most popular sports supplements.

## 2 Protein Supplementation

Protein supplementation is among the most commonly used sports supplements which aid athletes in achieving their target dietary intake of protein to maximize muscle gain [13]. Whey protein supplementation has been shown to improve muscle mass, strength, and exercise performance and is generally considered safe when consumed as recommended. However, excessive or prolonged use without appropriate medical or nutritional oversight has been associated with rare hepatic and renal adverse effects [14]. Whey is the liquid leftover of curdled milk after casein has been filtered out, which is subsequently dried up into protein powder. Unlike whole milk intake, whey supplements represent a highly concentrated, rapidly absorbed form of dairy protein that exerts potent metabolic and hormonal effects. Additionally, whey is rich in compounds that have been implicated in decreasing disease risk [13, 15].

Since its rise in popularity, studies ranging from case reports to clinical studies have been published linking consumption of these products to acneiform eruptions as seen in Figure 1. Milk, of which whey proteins are derived from, can aggravate acne by inducing increases in insulin and IGF‐1, and contributing to the release of estrogen, progesterone, and other comedogenic hormones [16, 17].

**FIGURE 1:**
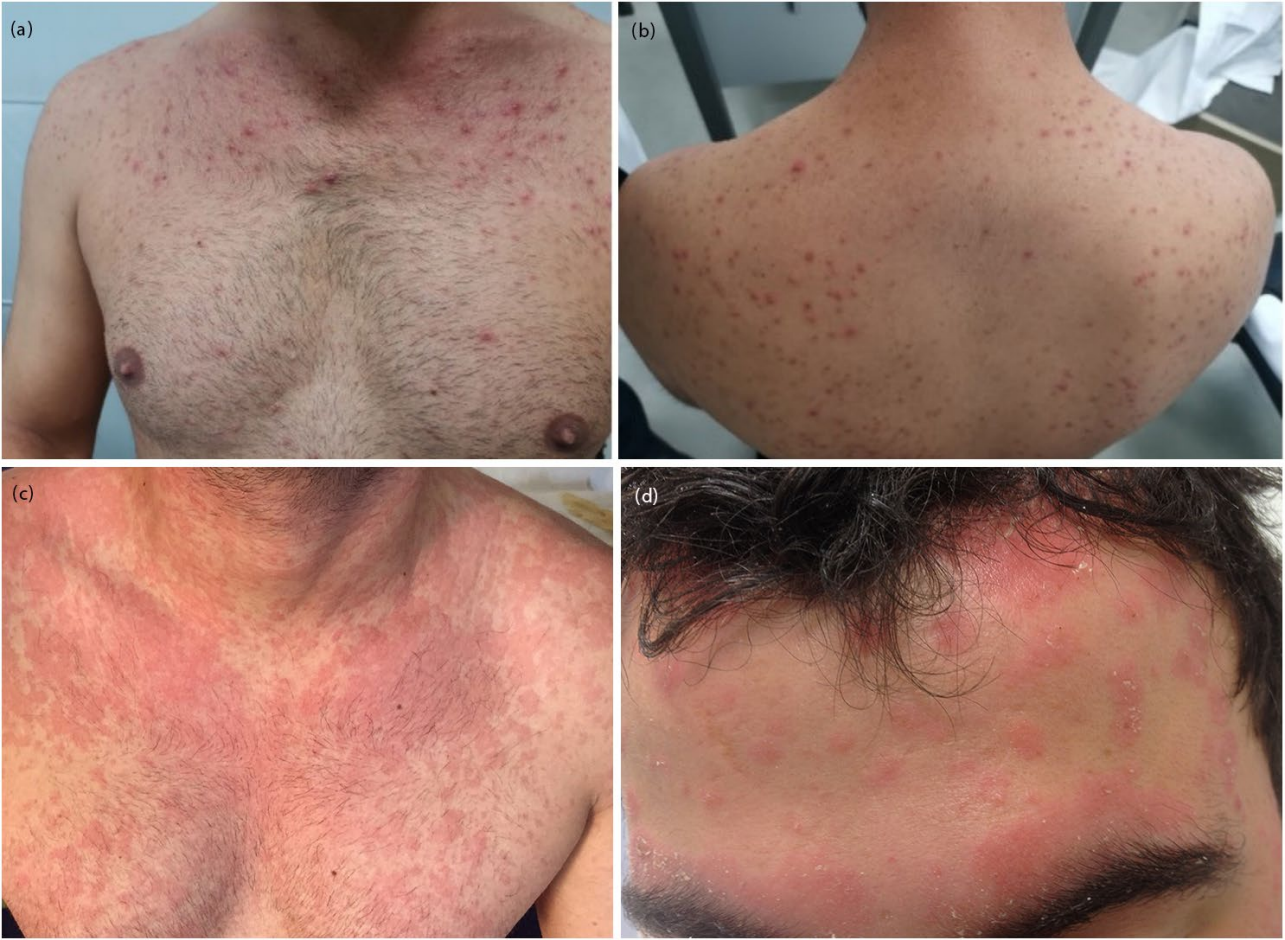
Various dermatologic diseases associated with whey protein. (a) and (b) acneiform eruptions over the chest and upper back; (c) tinea versicolor over the chest; and (d) seborrheic dermatitis over the forehead and frontal scalp.

Conflicted evidence regarding the potential relationship between acne and whey protein supplementation exists. Several published studies have explored the relationship between whey protein supplementation and acne, predominantly through small observational reports. Early evidence came from case series by Silverberg and Simonart, each describing five male patients who developed rapid‐onset acne shortly after initiating whey protein supplements [18, 19]. The former study reported complete resolution of acne after discontinuation of the whey protein, and recurrence of the condition in one patient after reintroduction of the supplement. Similarly, Cengiz et al. reported six male adolescents who developed prominent truncal acne during periods of rapid bodybuilding while consuming whey protein [20]. Pontes et al., in a prospective non‐controlled observational study, examined over a period of 60 days and over three instances, 30 individuals using protein‐calorie supplements [21]. Initially, 56.7% of the sample had acneiform lesions with degrees varying between I and II. At the end of the study, the entire sample had acneiform lesions with roughly a third of them presenting with degree III severity.

A case control published in 2024, based on the Jordanian population, investigated the relationship between whey protein supplementation and acne among 201 male adolescents and young adults attending fitness centers. Participants with acne (*n* = 100) were compared to acne‐free controls (*n* = 101) with similar demographics, body mass index, smoking status, and exposure to other known acne triggers including vitamin B12, corticosteroids, and anabolic steroids. The primary outcome assessed whey protein consumption within the preceding 3 months. Whey supplement use was significantly more common among acne patients (47%) than controls (27.7%) (*p* = 0.0047), and this association remained significant after multivariate adjustment. These findings provide supportive evidence that whey protein supplementation is independently associated with an increased risk of acne in young male athletes and supplement users. The study also reported that duration or frequency of intake did not correlate with acne severity [22].

Sompochpruetikul et al. conducted a double‐blind randomized controlled trial comparing 25 acne patients taking whey protein to 24 patients taking non‐whey protein supplements and found no significant difference in acne count or severity between the groups [23]. Forty‐nine participants were randomized to receive either 30 g of daily whey protein plus 18 g of a non‐whey nutritional supplement (WP group, *n* = 25) or 46 g of a non‐whey nutritional supplement (control group, *n* = 24) for 6 months. At follow‐up visits, investigators assessed total lesion counts, comedonal and inflammatory lesions, and overall acne severity. The mean age was comparable between groups (19.7 vs. 20.3 years). Changes in total facial and truncal lesion counts did not differ significantly between groups, with mean differences of −5.99 (95% CI −13.18–1.19, *p* = 0.09) for facial lesions and −2.18 (95% CI −11.83–7.48, *p* = 0.65) for truncal lesions. Only one participant (4.3%) in the non‐whey group experienced a notable worsening in Investigator Global Assessment score. Overall, the study concluded that whey protein supplementation did not result in greater worsening of acne lesion counts or severity compared with non‐whey supplementation over a 6 months period [23]. Collectively, while most early reports suggest a temporal association between whey supplementation and acne development, higher‐level evidence remains limited and somewhat conflicting.

Other dermatological manifestations may include seborrhea, androgenetic alopecia, and hirsutism, which may be related to the increased anabolic hormone response, which is linked to both muscle growth as well as the possible dermatological manifestations [24]. However, there is a paucity of data on these adverse events, and evidence is primarily anecdotal from patients. Some of these dermatologic manifestations are presented in Figure 1.

Besides acne, beneficial outcomes have been reported in animal models consuming whey protein. In a mouse model, mice were fed cow's milk casein (control), whey protein or casein protein hydrolysate (CPH) diets for 2 weeks prior to the induction of atopic dermatitis lesions. Mice fed with whey protein had significantly fewer lesions compared to those who were fed casein. Serum E‐selectin was lower in mice fed whey protein, a marker commonly increased in patients with AD [25]. Similarly, another study reported that diabetic mice supplemented with whey protein had enhancement in their inflammatory response to wound healing compared to diabetic mice that weren't supplemented with whey protein [26].

As for Branched Chained Amino Acids, which may be constituents of whey protein supplements or supplemented independently, they are commonly used by athletes to increase endurance and performance. Although some studies report no meaningful increases in these parameters, they are still commonly supplemented by athletes [27]. Chronic supplementation with BCAAs may negatively affect glucose metabolism and may lead to insulin resistance and Type 2 diabetes, and hence may display cutaneous signs of insulin resistance such as acanthosis nigricans and skin tags [28]. However, limited data exists on dermatological manifestations of BCAA supplementation. Acne may be considered a sign of insulin resistance and metabolic syndrome and may be expected in patients taking BCAAs [29].

## 3 Testosterone, DHEAS, and Anabolic Steroids Supplements

Despite their cutaneous and systemic side effects, anabolic steroid hormones remain commonly consumed by athletes with the intention of muscle growth and performance enhancement [30].

Acne vulgaris is a well‐documented adverse event of anabolic steroids, which may occur in previously unaffected individuals [31]. Both oral and intramuscular administration of AASs (Anabolic‐Androgenic Steroids) can induce the development of acne, which may be resistant to traditional therapies and persist long after the discontinuation of AASs [32, 33]. These supplements induce sebaceous gland hyperactivity, which, along with abnormal follicular keratinization, is implicated in the development of this disease [34]. Continued use may lead to severe acne variants such as acne conglobata and acne fulminans [35, 36, 37]. In similar pathophysiology, AASs stimulation of the sebaceous gland may lead to rosacea, seborrheic dermatitis, and epidermoid and sebaceous cysts [32, 38]. Sebaceous overactivity may lead to Tinea versicolor, dermatophyte skin infections, and recurrent folliculitis, and furunculosis may be possible as well. Androgenetic alopecia is also to be expected [32].

AASs can also lead to the formation of striae distensae secondary to the effect of AASs in increasing muscle mass and size, when the skin does not expand proportionate to the increase in muscle size [32, 38, 39].

Keloids have also been documented to develop in athletes during oral or IM use of AASs with no previous history of keloid formation. AASs have been implicated in decreasing the degradation and inducing production of Type 1 collagen, which may explain the development of these keloids [40, 41, 42].

AASs have also been known to affect hair growth, possibly leading to hirsutism or androgenetic alopecia which may persist even with the discontinuation of the supplement [32, 39]. AASs may also exacerbate pre‐existing skin conditions such as acne vulgaris. Reports of psoriasis exacerbation following AASs abuse, leading to psoriasis refractory to treatment, have been published [43]. A report of a male patient with familial angiolipomatosis experiencing an acute eruption of new tumors has been reported [44]. As well as a possible link between AASs use and the development of latent acute hereditary coproporphyria [45].

Other possible cutaneous adverse events from AASs use may be due to the hepatic injury of these agents, and the development of cutaneous sequelae of hepatic disease, or due to local injection site reactions from IM use of these agents [46].

### 3.1 Cutaneous Effects in Women

Data evaluating the dermatologic adverse effects of sports supplements in female athletes remain remarkably scarce. Most published studies investigating whey protein supplementation and anabolic‐androgenic steroid (AAS) use have enrolled predominantly male participants, limiting the generalizability of the current evidence. Women may experience distinct cutaneous manifestations owing to differences in endogenous hormonal milieu. In particular, exposure to AASs has been associated with virilizing effects including acne, hirsutism, female‐pattern androgenetic alopecia, menstrual irregularities, clitoromegaly, and irreversible voice deepening [47, 48]. Future prospective studies specifically evaluating female athletes are needed to better characterize the dermatologic effects and safety profile of sports supplements in women.

## 4 Other

Several other supplements have been consumed by athletes with the intention of improving sports performance and endurance. Beta‐alanine is a nonessential amino acid, commonly used for this purpose, has been reported to lead to paresthesias and itch [49, 50, 51].

Selenium, which is commonly used as a health supplement by the general population and athletes, has been reported to increase the risk of cutaneous melanoma [52]. Selenium, although it has essential functions for the thyroid and is an anti‐oxidant, has a narrow therapeutic window. Toxic doses of selenium, leading to selenosis, have profound dermatologic adverse events. Alopecia is often rapid and painless, occurring days to weeks after exposure. This is often followed by nail dystrophy and discoloration. It may also present with eczema, xerosis cutis [53].

Over supplementation with Niacin, often used in pre‐workout drinks and other energy drinks, may lead to a prostaglandin D‐2 mediated facial flushing, facial warmth, and erythema which is typically temporary; however, more severe cases have been reported along with erythroderma and an exfoliative dermatitis [54, 55].

High doses of Zinc supplementation, which have been an increasing trend in athletes, may cause a secondary copper deficiency, leading to hair loss and hypopigmented skin and hair lesions. Poor wound healing and chronic ulcers are possible as well [56, 57].

Supplementation with herbal products such as *Spirulina platensis, Aphanizomenon flos‐aqua, Chlorella, Echinacea, and alfalfa* has been reported to induce cutaneous autoimmune disorders such as dermatomyositis, several forms of lupus erythematosus, and pemphigus vulgaris and other blistering disorders [58]. Other cutaneous eruptions may be due to direct toxicity of ingesting supratherapeutic doses of supplements. Examples include skin desquamation and retinoid dermatitis from ingesting high doses of Vitamin A, cutaneous discoloration and yellowing of the skin from ingesting carotene derivatives, photosensitivity from ingesting high doses of pyridoxine. Methylcobalmin has been implicated in monomorphic acne eruptions and a case report of rosacea fulminans due to high dose supplementation with Vitamin B6 and B12 [59].

## 5 Conclusion

Overall, the use of sports supplements continues to increase among athletes and the general population. Consequently, healthcare professionals should be aware of the potential cutaneous adverse effects associated with these products (summarized in Table 1) to facilitate timely recognition, appropriate diagnosis, and management. However, the current evidence is largely limited to case reports and small observational studies, with relatively few controlled clinical investigations, precluding definitive conclusions regarding causality. Furthermore, an important limitation of the existing literature is the paucity of data involving female athletes, limiting the generalizability of current findings. A thorough medication and supplement history, including the timing of supplement initiation relative to symptom onset, is therefore essential when evaluating patients with suspected supplement‐related dermatoses. Future well‐designed prospective studies are needed to better characterize these associations and provide evidence‐based guidance for clinicians in this rapidly evolving field.

**TABLE 1:** Supplements and their associated purpose in athletes, reported dermatologic diseases, and proposed mechanisms behind these diseases.

Supplement/Category	Purpose in athletes	Proposed mechanism	Reported dermatologic manifestations
Whey protein	Increase muscle mass, strength, and performance; help meet protein intake	Increases insulin and IGF‐1; hormonal changes (estrogen, progesterone, androgen signaling) that may stimulate sebaceous activity	Acneiform eruptions; possible seborrhea, androgenetic alopecia, hirsutism (limited evidence)
Branched‐chain amino acids (BCAAs)	Improve endurance and athletic performance	May impair glucose metabolism and contribute to insulin resistance	Possible acne, acanthosis nigricans, skin tags (signs of insulin resistance)
Anabolic androgenic steroids (AASs)	Muscle growth and performance enhancement	Sebaceous gland hyperactivity, androgen stimulation, altered collagen metabolism	Acne vulgaris (often severe), acne conglobata/fulminans, seborrhea, rosacea, seborrheic dermatitis, cysts, infections, androgenetic alopecia, hirsutism, striae distensae, keloids
AASs – Other skin associations	Same as above	Hormonal and immune modulation; systemic effects including hepatic injury	Psoriasis exacerbation, folliculitis, furunculosis, tinea versicolor, injection site reactions, cutaneous signs of liver disease
Beta‐alanine	Improve endurance and exercise performance	Neurologic stimulation affecting sensory nerves	Paresthesia, pruritus
Selenium (excess)	General health/antioxidant supplementation	Toxicity (selenosis) affecting hair follicles and skin	Rapid alopecia, nail dystrophy, eczema, xerosis; possible melanoma risk
Niacin (high doses)	Used in energy/pre‐workout supplements	Prostaglandin D2–mediated vasodilation	Flushing, erythema, warmth; severe cases: erythroderma, exfoliative dermatitis
Zinc (excess)	Immune and performance supplementation	Secondary copper deficiency	Hair loss, hypopigmented skin/hair lesions, poor wound healing, ulcers
Herbal supplements (e.g., Spirulina, Chlorella, Echinacea, alfalfa)	Performance and health supplements	Immune activation/autoimmune triggering	Dermatomyositis, lupus erythematosus variants, pemphigus vulgaris, blistering disorders
Vitamin Over‐supplementation (A, carotene, B6, B12)	Performance or energy supplementation	Direct toxicity or metabolic effects	Desquamation, retinoid dermatitis (Vit A), yellow skin discoloration (carotenemia), photosensitivity (B6), monomorphic acne or rosacea fulminans (B6/B12)

## Funding

The authors have nothing to report.

## Disclosure

Use of Artificial Intelligence (AI) Statement: Generative AI was not used in the write‐up or development of the manuscript.

## Conflicts of Interest

The authors declare no conflicts of interest.

## Data Availability

Data sharing not applicable to this article as no datasets were generated or analysed during the current study.
